# Long non-coding RNA lnc-CHAF1B-3 as a new player in fibrosis

**DOI:** 10.1016/j.omtn.2023.02.013

**Published:** 2023-02-28

**Authors:** Ilayda Sen, Shizuka Uchida, Venkata Naga Srikanth Garikipati

**Affiliations:** 1 Department of Emergency Medicine, The Ohio State University Wexner Medical Center, Columbus, OH 43210, USA; 2 Center for RNA Medicine, Department of Clinical Medicine, Aalborg University, Frederikskaj 10B, 2. (building C), 2450 Copenhagen SV, Denmark; 3 Dorothy M. Davis Heart Lung and Research Institute, The Ohio State University Wexner Medical Center, Columbus, OH 43210, USA

**Figure 1:**
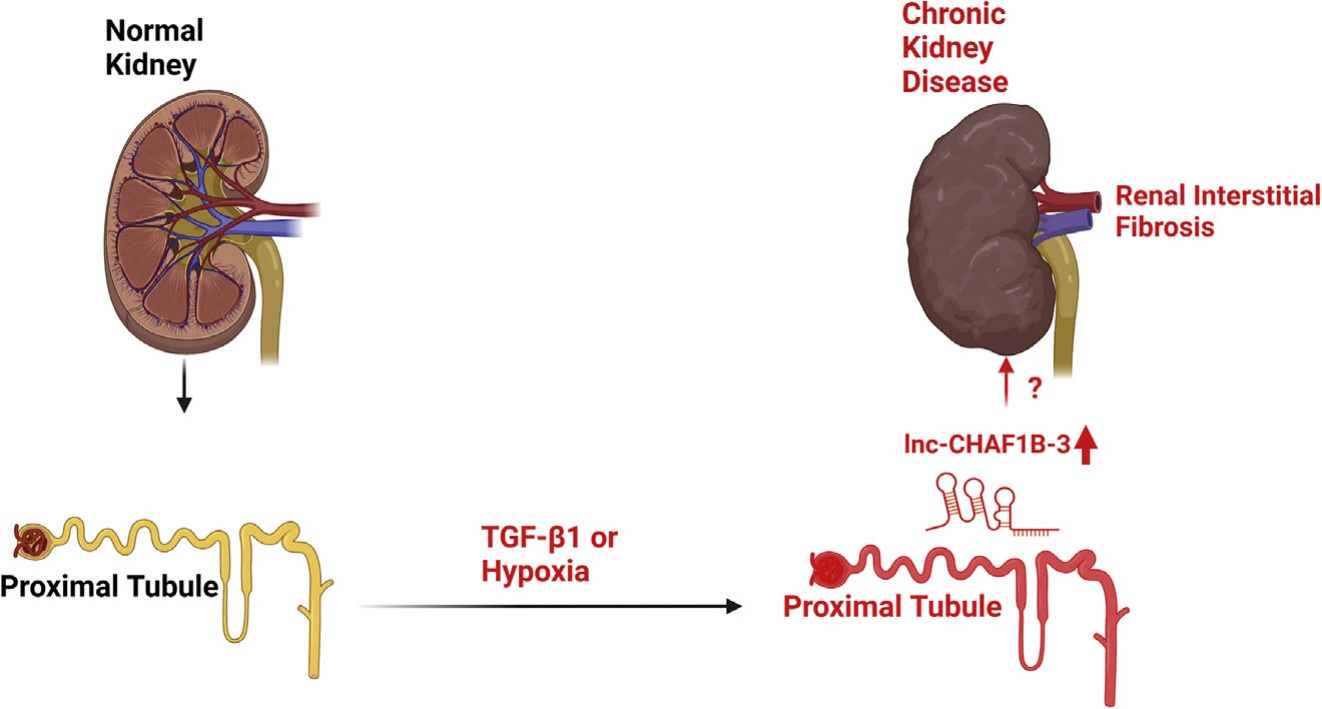
The scheme represents the role of lnc-CHAF1B-3 potentially involved in renal fibrosis and chronic kidney disease Figure was made using Biorender.

Chronic kidney disease (CKD) is one of the leading causes of death worldwide. It affects more than 10% of the global population.^1^ The end stage of CKD results in renal intestinal fibrosis (RIF), which causes renal dysfunction. The mechanism behind RIF is largely unknown. As such, there is no effective treatment for this condition.^2^^,^^3^ Given the increasing incidence of RIF and CKD, it is of the utmost importance to understand the fundamental molecular basis of RIF, which may lead to the development of both preventive and treatment options for patients with CKD. Along these lines, the study by Imai et al.^4^ in this issue of *Molecular Therapy – Nucleic Acids* provides a new avenue to the potential role of the long non-coding RNA (lncRNA) *lnc-CHAF1B-3* in the development of human kidney disease **(**Figure 1).

The long-standing assumption that the non-coding regions of RNAs (ncRNAs) are mostly non-functional has been challenged in recent years due to emerging research unveiling their likely ability to act as therapeutic targets or diagnostic biomarkers for a wide variety of human diseases. lncRNAs are a subtype of ncRNAs that are longer than 200 nucleotides. They are increasingly recognized as regulators of gene expression in both physiological and pathological conditions.^5^ Recent studies have uncovered that lncRNAs may serve as diagnostic biomarkers in the serum/plasma samples derived from patients with renal fibrosis.^6^ However, the role of lncRNAs in the progression of RIF remains obscure and is the focus of this new study.^4^ Here, the authors comprehensively investigated the lncRNA signature in the context of renal fibrosis using unbiased microarray analysis. To achieve this, the authors utilized two types of human renal tubular epithelial cells: immortalized human proximal tubular cells (HK2) and primary cultured human renal proximal tubular epithelial cells (RPTECs). These cells were treated with TGF-β1 or hypoxia, which are key mediators of renal fibrosis in CKD. Results revealed about 21,470 lncRNAs were differentially expressed (either up- or down-regulated) in both cell types subjected to TGF-β1 or hypoxia stress. Interestingly, in both stressed conditions in both cell types, five lncRNAs (*lnc-CHAF1B-3*, *lnc-CHAF1B-2*, *MIR181A2HG*, *LOC100507487*, and *LINC01638*) were commonly upregulated. Based on lncRNA expression levels and the unknown function of *lnc-CHAF1B-3*, the authors chose to investigate this lncRNA further.

To understand the physiological role of *lnc-CHAF1B-3*, Imai and colleagues^4^ silenced *lnc-CHAF1B-3* using both small interfering RNA (siRNA) and antisense oligos in two types of epithelial cells, HK2 and RPTECs, with or without TGF-β1 stimulation. Results from these experiments revealed robustly suppressed TGF-β1-induced upregulated expression of epithelial-mesenchymal transition (EMT)-related signaling in renal tubular epithelial cells, emphasizing the significance of *lnc-CHAF1B-3* in the development of renal fibrosis. From a translational perspective, the authors further investigated the expression levels *of lnc-CHAF1B-3* in patients with immunoglobulin A (IgA) nephropathy (chronic kidney disease) in five mild and 13 advanced cases. Intriguingly, RT-PCR analysis of the masked-form fixed paraffin-embedded kidney biopsies from the aforementioned patients revealed significantly elevated *lnc-CHAF1B-3* levels in patients with advanced compared with minor glomerular abnormality (MGA) present with preserved renal function and a urinary protein similar to mild cases of IgA nephropathy, which were chosen as controls. Most importantly, Spearman’s correlation analysis revealed a positive correlation among *lnc-CHAF1B-3*, urinary protein concentrations, and the progression of renal dysfunction in patients with IgA nephropathy. To further determine the localization of *lnc-CHAF1B-3*, authors performed segment-specific multiplexed fluorescent *in situ* hybridization (FISH) assays on kidney biopsy samples, where CD10 and epithelial membrane antigen were used to label proximal tubules and distal tubules, respectively. FISH results revealed that *lnc-CHAF1B-3* localized only to the cytoplasm of the proximal tubules but not in fibroblasts, irrespective of disease severity. However, further work will also be required to determine if the proximal tubule’s specific inhibition of *lnc-CHAF1B-3* inhibits renal fibrosis and renal dysfunction in a suitable *in vivo* model, such as non-human primates or pigs as the sequence conservation is higher in these large animals compared with rodents. Taken together, these results highlight the potential clinical implications of *lnc-CHAF1B-3* in CKD.

It should be noted that although there are two isoforms of *lnc-CHAF1B-3* (*CLDN14-AS1-201* [Ensembl Transcript ID, ENST00000428667.1] and *CLDN14-AS1-202* [ENST00000454980.1]) according to the latest annotation provided by the Ensembl database, the authors did not discuss which isoform was investigated. Therefore, given that the genomic locus of this lncRNA is complicated with the presence of a novel transcript (Ensembl Gene ID, ENSG00000230479) and the protein-coding gene, claudin 14 (*CLDN14*), in the antisense direction, future studies are required to perform a 5/3′ rapid amplification of cDNA ends (RACE) assay to obtain the full-length sequence of each isoform of *lnc-CHAF1B-3* to pinpoint the target lncRNA transcript. Furthermore, the authors attempted to decipher the mechanism of action of *lnc-CHAF1B-3* by exploring lncRNA-microRNA (miRNA) interactions that drive *lnc-CHAF1B-3*-mediated EMT signaling, and it was revealed that *lnc-CHAF1B-3* does not act as an miRNA sponge. Since *lnc-CHAF1B-3* is localized in the cytoplasm of the proximal tubules, future work is warranted to tease out molecular mechanisms and see if *lnc-CHAF1B-3* regulates EMT signaling through post-transcriptional modifications. Nevertheless, the current study by Imai and colleagues^4^ provides important new information to the active lncRNA field by identifying *lnc-CHAF1B-3* in human RIF.
